# Cost-utility of medication withdrawal in older fallers: results from the improving medication prescribing to reduce risk of FALLs (IMPROveFALL) trial

**DOI:** 10.1186/s12877-016-0354-7

**Published:** 2016-11-04

**Authors:** Suzanne Polinder, Nicole D. A. Boyé, Francesco U. S. Mattace-Raso, Nathalie Van der Velde, Klaas A. Hartholt, Oscar J. De Vries, Paul Lips, Tischa J. M. Van der Cammen, Peter Patka, Ed F. Van Beeck, Esther M. M. Van Lieshout, the IMPROveFALL trial collaborators, T. J. M. van der Cammen, P. Patka, E. F. Van Beeck, N. Van der Velde, E. M. M. Van Lieshout, S. Polinder, F. U. S. Mattace-Raso, K. A. Hartholt, N. D. A. Boyé, C. W. N. Looman, P. Lips, O. J. De Vries, A. J. H. Kerver, M. M. M. Bruijninckx, M. R. de Vries, G. Ziere

**Affiliations:** 1 Department of Public Health, Erasmus MC, University Medical Center Rotterdam, PO Box 2040, 3000 CA Rotterdam, The Netherlands; 2 Section of Geriatric Medicine, Department of Internal Medicine, Erasmus MC, University Medical Center Rotterdam, Rotterdam, The Netherlands; 3 Trauma Research Unit Department of Surgery, Erasmus MC, University Medical Center Rotterdam, Rotterdam, The Netherlands; 4 Department of Emergency Medicine, Erasmus MC, University Medical Center Rotterdam, Rotterdam, The Netherlands; 5 Department of Internal Medicine, VU University Medical Center, Amsterdam, The Netherlands

**Keywords:** Quality of life, Medication withdrawal, Cost utility, Older persons, Falls

## Abstract

**Background:**

The use of Fall-Risk-Increasing-Drugs (FRIDs) has been associated with increased risk of falls and associated injuries. This study investigates the effect of withdrawal of FRIDs versus ‘care as usual’ on health-related quality of life (HRQoL), costs, and cost-utility in community-dwelling older fallers.

**Methods:**

In a prospective multicenter randomized controlled trial FRIDs assessment combined with FRIDs-withdrawal or modification was compared with ‘care as usual’ in older persons, who visited the emergency department after experiencing a fall. For the calculation of costs the direct medical costs (intramural and extramural) and indirect costs (travel costs) were collected for a 12 month period. HRQoL was measured at baseline and at 12 months follow-up using the EuroQol-5D and Short Form-12 version 2. The change in EuroQol-5D and Short Form-12 scores over 12 months follow-up within the control and intervention groups was compared using the Wilcoxon Signed Rank test for continuous variables and the McNemar test for dichotomous variables. The change in scores between the control and intervention groups were compared using a two-way analysis of variance.

**Results:**

We included 612 older persons who visited an emergency department because of a fall. The mean cost of the FRIDs intervention was €120 per patient. The total fall-related healthcare costs (without the intervention costs) did not differ significantly between the intervention group and the control group (€2204 versus €2285). However, the withdrawal of FRIDs reduced medication costs with a mean of €38 per participant. Furthermore, the control group had a greater decline in EuroQol-5D utility score during the 12-months follow-up than the intervention group (*p* = 0.02). The change in the Short Form-12 Physical Component Summary and Mental Component Summary scores did not differ significantly between the two groups.

**Conclusions:**

Withdrawal of FRID’s in older persons who visited an emergency department due to a fall, did not lead to reduction of total health-care costs. However, the withdrawal of FRIDs reduced medication costs with a mean of €38 per participant in combination with less decline in HRQoL is an important result.

**Trial registration:**

The trial is registered in the Netherlands Trial Register (NTR1593 – October 1^st^ 2008).

**Electronic supplementary material:**

The online version of this article (doi:10.1186/s12877-016-0354-7) contains supplementary material, which is available to authorized users.

## Background

Fall incidents represent an increasing burden on health care systems in aging societies worldwide. Falls affect a large proportion of persons aged 65 years and older and are associated with high mortality and morbidity, leading to great personal suffering, represented in loss of quality of life and high costs [1–5]. Older fallers cause high numbers of Emergency Department (ED) visits and hospital admissions [6, 7]. In 2000, the fall-related medical costs in the population aged 65 years and older in the United States amounted to US$19 billion for non-fatal injuries and US$200 million for fatal injuries [8]. Between 2003 and 2007 the average annual cost for fall-related injuries in the Netherlands was US$640 million (€470 million) [9]. The overall cost per fall was US$10,540 (€7800), mainly caused by direct medical costs [10].

In order to reduce the prevalence of falls, potentially avoidable risk factors have been well documented [11–13], and there has been a substantial number of falls prevention trials [5, 14–22]. However, past variations in outcome definitions and measures of falls prevention trials have hindered comparative research and meta-analysis, and thus the *Prevention of Falls Network Europe* (ProFaNE) established a common set of outcome definitions and measures for use in trials. These include costs, health-related quality of life (HRQoL) outcomes, and a follow-up duration of 12 months [23]. But so far only few fall-prevention trials have documented quality of life outcomes [18, 24–27] and the HRQoL as recommended by ProFaNE has been reported in only one of them [18]. Moreover, economic evaluations on falls prevention are scarce. However, evidence from reviews targeting economic evaluation studies of single factor falls prevention interventions like exercise programs [28, 29] and multifactorial falls prevention interventions [30] is promising.

The use of FRIDs has been associated with increased risk of falls and associated injuries [31–35]. The withdrawal of FRIDs has been shown to be safely possible and to generate significant cost savings in some patients [14, 36, 37], but the cost-utility of this approach has not been reported yet. The present study investigated costs, the effect on HRQoL, and the cost-utility of a structured medication assessment including withdrawal of FRIDs versus ‘care as usual’ in community-dwelling older men and women, who visited the ED after experiencing a fall [38].

## Methods

### Study design

The IMPROveFALL study is a multicenter randomized controlled trial in the Netherlands. Eligible patients were randomized to one of the treatment arms using a web-based randomization program. Variable block randomization was accomplished via a trial website. Allocation was at random and concealed. The patients were randomized to the intervention group or ‘care as usual’. It was not possible to blind the geriatrician and patients for the allocation of the study group. The study was performed in accordance with the Declaration of Helsinki and all participants gave written informed consent. The local Medical Research Ethics Committees in the six participating hospitals approved the study protocol. A detailed description of the study protocol can be found elsewhere [38].

### Study population

Patients meeting the following inclusion criteria were eligible for enrolment: age 65 years or older, visited the ED due to a fall, use of one or more FRIDs [32, 33, 35, 38], Mini-Mental State Examination (MMSE) score of at least 21 out of 30 points [39], ability to walk independently, community dwelling, and provision of written informed consent by the patient. Participating hospitals included two academic and four regional hospitals in the Netherlands. Enrolment started in October 2008 and was completed in October 2011. The follow-up period was 12 months.

All persons visiting the ED because of a fall received care as usual for their injuries. Following the ED visit, patients were contacted by telephone. Subsequently, eligible and interested potential study participants received an appointment for the research outpatient clinic. The visits to the research outpatient clinic took place within two months after the fall-related ED visit. If the patient met all eligibility criteria, the patient was asked to sign the Informed Consent form. During the visit to the research outpatient clinic, a fall-related assessment was performed by the research physician.

### Intervention

All participants received a structured medication assessment. The intervention group consisted of a systematic FRIDs assessment combined with FRIDs withdrawal or modification, if safely possible. A complete list of FRIDs is presented in Appendix A. For each drug, the research physician assessed whether the initial indication still existed. Proposed changes in medication were discussed with a senior geriatrician, and if necessary with the prescribing physician. A research nurse offered counselling, evaluated possible negative effects via a standardized telephone follow-up, and discussed any problems regarding the drug modification with the research physician and geriatrician.

### Definition fall incident

A fall was defined as coming to rest unintentionally on the ground or a lower level with or without losing consciousness, but not induced by acute medical conditions, e.g., stroke, or exogenous factors such as a traffic accident [40]. All participants received a Falls Calendar for reporting falls during a one-year follow-up period. Falls were recorded weekly on the Fall Calendars, which had to be returned every three months. Follow-up started two weeks after completed intervention or two weeks after initial research clinic visit when no intervention was performed.

### Costs

The total direct and indirect costs of both FRIDs withdrawal and ‘care as usual’ were measured. Costs were calculated by multiplying the volumes of healthcare use with the corresponding unit prices (Table 1). Direct healthcare costs included the costs of the FRIDs assessment and modification, drug consumption (i.e., the cost of substitution drugs), and fall-related healthcare consumption during one year of follow-up (e.g., outpatient visits, hospital admissions, General Practitioner consultations, home care, nursing home care). Indirect costs included patient travel costs. For the intervention (systematic fall-related drugs assessment) the full cost price was calculated and for the other healthcare costs standard Dutch cost prices were used as published earlier by *Hakkaart-van Roijen* et al. [41]. Costs of medication use were recorded in the study, and unit costs were determined with information from the National Dutch Formulary [42]. Healthcare consumption, both fall and non-fall related, and patient costs were recorded from the three-monthly questionnaires for healthcare consumption and patient costs. Furthermore we collected data from the participants’ General Practitioner, by sending a questionnaire on healthcare use.

**Table 1:** List of costs

Cost categories	Parameter	Source of consumption data	Cost price (€, 2009)
Intervention costs	*	Study registry	Variable
Medication costs	DDD	Study registry	Variable
Hospital stay costs	Day	Hospital registry	457
Emergency Department costs	Visit	Hospital registry	151
General Practitioner costs	Consultation	Questionnaire	28
Specialist consult costs	Consultation	Hospital registry	72
Home care costs	Per hour	Questionnaire	35
Physical therapy costs	Visit	Questionnaire	36
Nursing home costs	Day	Questionnaire	238
Intermediate care facility costs	Day	Questionnaire	90
Rehabilitation center costs	Day	Questionnaire	340
Patient costs (travel costs)	Per kilometer	Questionnaire	Variable**

*DDD* Defined Daily Dose, *GP* General Practitioner

*Geratric consultation (€72) + routine blood test (€20) + extra consults (€72)

**Private motor vehicle/public transportation/taxi

The number of injuries prevented was calculated with data recorded in the three-monthly questionnaire, supplemented with epidemiological data on falls and injury risks. These were supplemented with data on healthcare costs of injury from previous research [10].

### Health-related quality of life

During the baseline assessment and during the follow-up visit at 12 months follow-up, all participants were asked to complete the patient outcome questionnaire (see Additional file 1), under supervision of the clinical investigator or research nurse. Based upon the recommendations of ProFaNE [23], HRQoL was measured using the Dutch versions of the EuroQol-5D (EQ-5D) [43] and the Short Form-12 (SF-12) version 2 [44]. The EQ-5D is recommended for the assessment of HRQoL in trauma patients, especially for economic assessments [45]. The EQ-5D instrument covers five health domains (mobility, self-care, usual activities, pain/discomfort, and anxiety/depression). Each dimension has three levels; no problem, moderate problem, or severe problem. In addition, a scoring algorithm based on empiric valuations from the United Kingdom general population and subsequent statistical modeling is available by which the health status descriptions can be expressed into a utility score [46]. This utility score ranges from 1 for full health to 0 for death, and can be interpreted as a judgment on the relative desirability of a health status compared with perfect health. The EQ-5D is a validated and extensively used general health questionnaire to measure quality of life [43]. The SF-12 contains eight domains measuring physical and mental health outcomes; physical functioning, role physical, bodily pain, general health, vitality, social functioning, role emotional, and mental health. Data from all eight domains are used to construct the physical and mental component summary measures (PCS and MCS) [44].

### Cost-utility analysis

The long-term effectiveness of the interventions was expressed in terms of the cumulative number of life years and quality-adjusted life years (QALYs) gained. The QALY combines morbidity and mortality into a single number. QALYs were calculated by weighting life years for the quality of life using the EQ-5D utility score over 12 months (12 months follow-up minus baseline). The gain in QALY is equal to the difference of QALY outcomes between the two study arms.

Finally, the cost per QALY gained was calculated as the ratio of total intervention costs minus savings in fall-related healthcare costs compared with control divided by the cumulative QALYs gained compared with control. All analyses were performed in accordance with Dutch guidelines for economic evaluations [47].

### Statistical analyses

All analyses were performed using the Statistical Package of the Social Sciences (SPSS version 17.0, Chicago, Ill.) and a *p*-value < 0.05 was considered statistically significant. Missing data were not imputed. Baseline characteristics were compared using Student *t*-test analyses for continuous variables and chi-squared analyses for dichotomous variables. The change in EQ-5D utility score and SF-12 PCS and MCS scores over 12 months (i.e., after 12 months follow-up minus baseline data) within the control and intervention groups were compared using the Wilcoxon Signed Rank test for continuous variables and the McNemar test for dichotomous variables. The change in scores between the control and intervention groups were compared using a two-way analysis of variance (ANOVA). Analyses of the individual health domains of the EQ-5D and SF-12 were also performed. Secondary analyses were performed, comparing the HRQoL scores of the participants with and without a fall during follow-up.

## Results

**Fig. 1:**
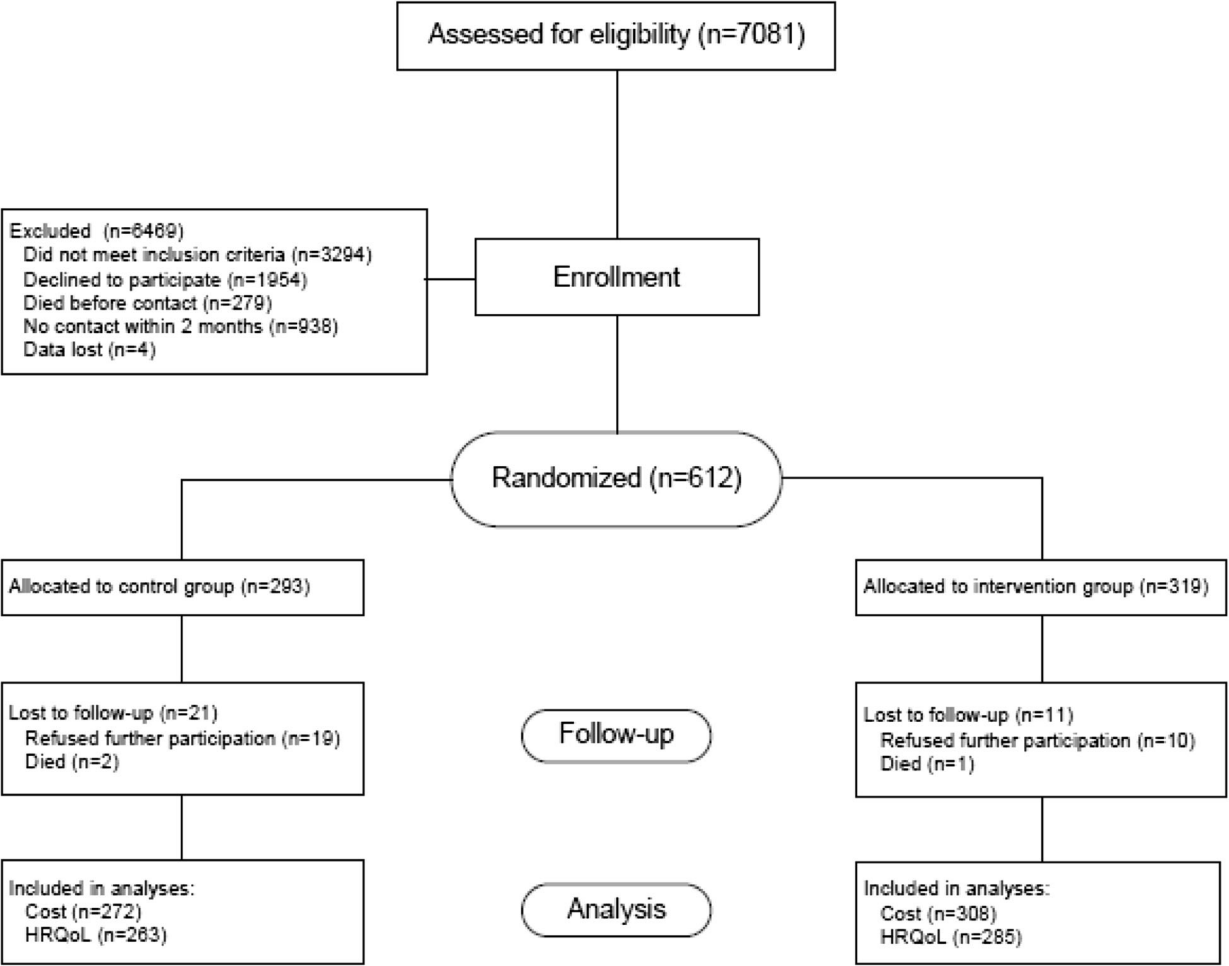
Flowchart of study participants. *Of the participants who died during follow-up, most were included in the analyses, except for two in the control and one in the intervention group. **Nine and 23 participants in the control and intervention group declined or were unable to complete EQ-5D questionnaires after 12-months follow-up

In total, 7081 ED visitors were screened for inclusion in the study, of which 3294 were not eligible, 1954 refused to participate, 279 persons died before contact, 938 patients were failed to contact within 2 months, and of 4 patients data was lost. Subsequently, 612 participants were randomized in the IMPROveFALL study (Fig. 1).

This randomization resulted in 319 participants to the intervention group and 293 participants allocated to the control group. In total 271 participants from the control group and 308 participants from the intervention group had complete intramural and extramural cost information. Finally, 265 and 287 participants respectively from the control and intervention group completed quality of life assessments at baseline and at 12 months follow-up (Fig. 1). The mean age was 76 years and 62 % of the study population was female. No significant differences in baseline characteristics were found between the control and intervention group (Table 2).

**Table 2:** Baseline characteristics of the control and intervention group

	Control *n* = 293	Intervention *n* = 319
Demographics		
Age (year)	76.4 ± 6.6	76.5 ± 7.2
Female gender	182 (62)	198 (62)
MMSE	27.0 ± 2.4	27.0 ± 2.3
BMI (m^2^/kg)	27.6 ± 4.7	27.6 ± 4.6
Fall risk factors		
Charlson Comorbidity Index	1.9 ± 1.6	1.9 ± 1.6
Number of drugs	6.4 ± 3.3	6.3 ± 3.3
Number of FRIDs	3.9 ± 2.0	3.9 ± 2.1
History of recurrent falls	128 (44)	148 (46)
Smoking	37 (13)	34 (11)
Alcohol intake (≥3 units/day)	33 (11)	34 (11)
Functional status		
Home care	69 (24)	82 (26)
Activities of Daily Living	0.80 ± 4.5	0.80 ± 3.3
Instrumental Activities of Daily Living	1.39 ± 5.4	1.37 ± 4.0

Continuous data are shown as mean values ± standard deviation, categorical data as number with percentage

*MMSE* Mini-Mental State Examination, *BMI* Body Mass Index, *FRID* Fall-Risk Increasing Drugs

The number of participants in the control group and intervention group experiencing a fall or recurrent fall during the one-year follow-up did not differ significantly (34 % versus 37 %; *p* = 0.33) [48].

The mean number of FRIDs used at baseline was four. In 40 % of all FRIDs an intervention was deemed not possible or not necessary. Of all attempted FRID-withdrawals 35 % failed, either due to non-compliance or due to a return of the primary indication for which the drug had initially been prescribed. More detailed specifications on the interventions according to FRID categories and specific drug types, and details on compliance to attempted interventions has been published before [48].

### Intervention costs

The mean cost of the FRIDs intervention was €120 per patient, which included the initial research clinic assessment (€72), routine blood tests (€20) and when necessary (78 patients one or more checks) additional checks/assessments (€72).

### Cost savings

The mean costs saved with medication withdrawal, dose reduction and drug substitution was €38 per participant for the intervention group.

For all other intramural and extramural care no significant differences in costs were found, except for general practitioner visits (Table 3). However, for all but two health care items the costs were lower for the intervention group. Rehabilitation caused relatively the highest costs, with much higher costs for the intervention group than for the control group (€708 versus €229; NS). In total 15 patients received rehabilitation care (10 in the intervention arm) varying from 6 to 120 days stay, of which the 6 patients with the longest stay in rehabilitation were all in the intervention arm. Comparing intermediate care facility and nursing home costs between both groups, it is striking that the numbers of patients receiving either type of care were similar, however, the long stayers for both health care facilities were in the control group.

**Table 3:** Mean costs per patient of the control and intervention group during 12 months follow-up

Cost categories	Control (*n* = 272)	Intervention (*n* = 308)	*p*-value
Intervention costs	-	120^†^	*
General Practitioner consult costs	29	20	*
Specialist consult costs	51	40	
Emergency Department costs	12	10	
Hospital stay costs	360	383	
Home care costs	662	630	
Physical therapy costs	290	218	
Intermediate care facility costs	220	74	
Nursing home costs	424	156	
Rehabilitation center costs	229	708	
Patient costs (travel costs)	3	2	
Change in medication costs^‡^	−3	−38	*
Total costs	2285	2324	

Data are given as mean values in euro (€).^†^Average;* < 0.05

‡ The change in medication costs was reported, since the main aim of the intervention was to withdraw medication. The total costs of medication in general is related to the health state and comorbidity at start of the intervention and were highly driven by some outliers

The intramural and extramural fall-related healthcare costs (without the intervention costs of €120) did not differ significantly between the intervention group compared with the control group (€2204 versus €2285; NS).

### Health-related quality of life

Nine participants in the control and 23 in the intervention group declined or were unable to complete the EQ-5D questionnaires after 12-months follow-up. Additional 5 and 2 participants in the control and intervention group, respectively, had incomplete SF-12 questionnaires after 12-months follow-up.

**Fig. 2:**
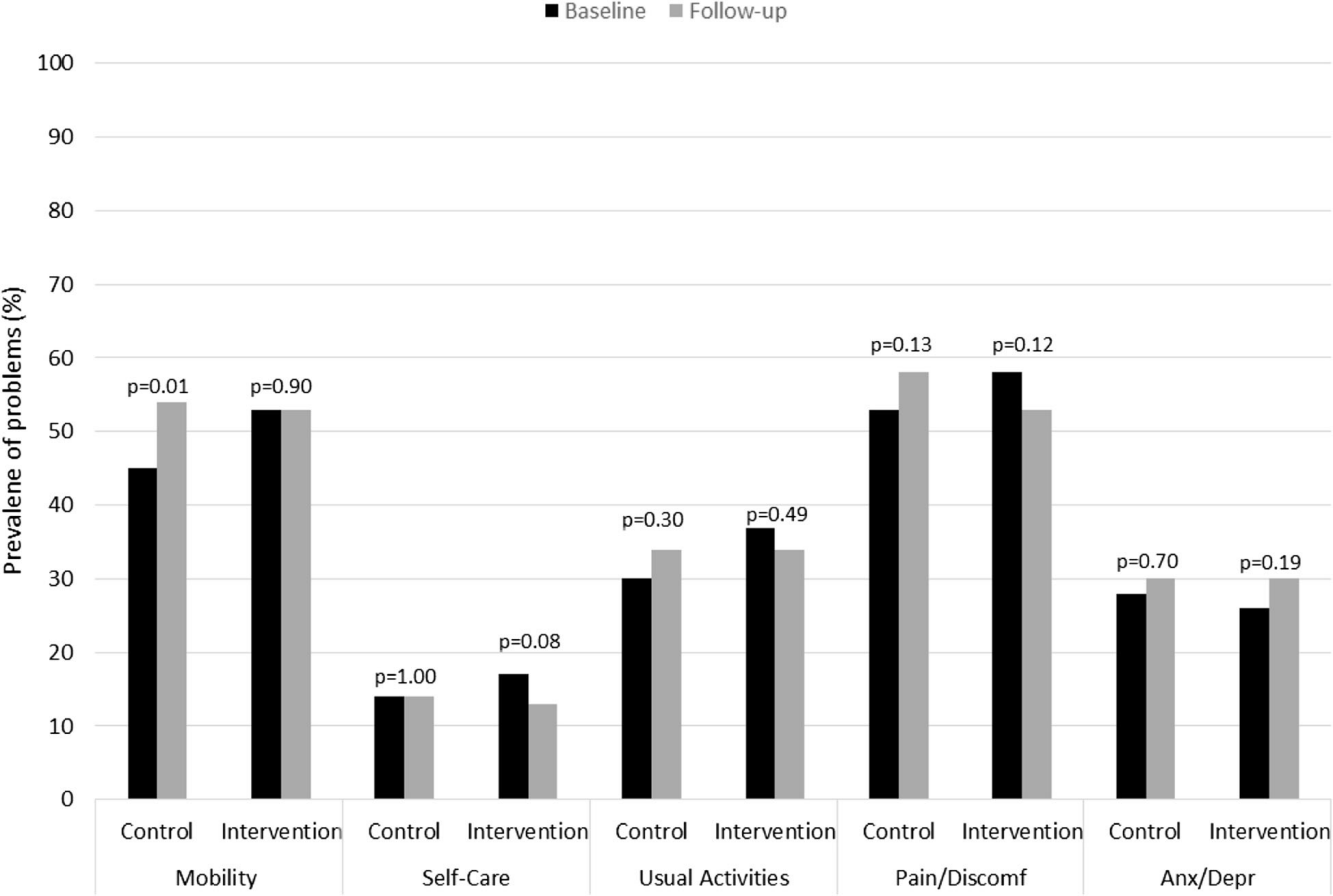
Prevalence of problems on the five dimensions of the EQ-5D in the control and intervention groups at baseline and 12 months follow-up. Nine and 23 participants in the control and intervention group declined or were unable to complete EQ-5D questionnaires after 12-months follow-up. *p-values were measured with the McNemar test

The baseline and follow-up HRQoL scores of the control and intervention group are shown in Table 4. The control group had a greater decline in EQ-5D utility score during the 12-months follow-up than the intervention group, (*p* = 0.02). The decline in the SF-12 PCS and MCS score did not differ significantly between the two groups (*p* = 0.08 and *p* = 0.90). The problems in the EQ-5D domains of the control and intervention group reported at baseline and at follow-up are shown in Fig. 2. Control patients reported significantly more problems with mobility (increase of 9 %; *p* = 0.01) at 12-months follow-up, which mainly explains the decline in HRQoL in the control group.

**Table 4:** Quality of life scores of the control and intervention group at baseline and 12 months follow-up, and the change over 12 months

	Group	N^†^	Baseline	Follow-up	*p*-values*	Change	*p*-values**
EQ-5D utility score	Control	263	0.78 ± 0.22	0.74 ± 0.25	0.01	−0.04 ± 0.22	0.02
	Intervention	285	0.74 ± 0.26	0.75 ± 0.26	0.75	0.01 ± 0.24	
SF-12 PCS score	Control	258	46.2 ± 9.9	42.2 ± 11.6	<0.01	−3.9 ± 8.5	0.08
	Intervention	283	45.6 ± 9.5	43.0 ± 10.7	<0.01	−2.6 ± 8.5	
SF-12 MCS score	Control	258	53.2 ± 9.0	52.5 ± 9.2	0.28	−0.7 ± 9.7	0.90
	Intervention	283	53.3 ± 9.5	52.5 ± 9.0	0.20	−0.8 ± 9.7	

Data are given as mean values ± standard deviation

^†^9 and 23 participants in the control and intervention group declined or were unable to complete EQ-5D questionnaires after 12-months follow-up, an additional 5 and 2 participants in the control and intervention group had incomplete SF-12 questionnaires after 12-months follow-up

*Wilcoxon Signed Rank test (comparing baseline and follow-up)

**Two-way ANOVA of the change over 12 months

The overall mean baseline EQ-5D utility score of participants with and without a fall during follow-up was 0.69 ± 0.27 and 0.80 ± 0.21, respectively (*p* < 0.01). The overall mean baseline SF-12 PCS scores of those with and without a fall during follow-up were 44.4 ± 9.9, and 46.6 ± 9.6, *p* = 0.01. The overall mean baseline SF-12 MCS scores of those with and without a fall during follow-up were 53.2 ± 10.0, and 53.3 ± 9.0, *p* = 0.87. Thus, the participants who experienced further falls during follow-up had significantly lower EQ-5D and SF-12 PCS scores at baseline. A secondary analysis was performed of the decline in HRQoL in the participants of the control and intervention group with and without a fall during follow-up (Table 5). In the participants with a fall during follow-up, the change in quality of life did not differ significantly between both groups. In the participants without a fall during follow-up, the control group had a greater decline in the SF-12 PCS score (*p* = 0.01) than the intervention group.

**Table 5:** Quality of life scores of the participants with and without a fall during follow-up

Fall	Group	N	Baseline	Follow-up	*p*-values*	Change	*p*-values**
EQ-5D utility score	Control	87	0.71 ± 0.25	0.64 ± 0.28	0.01	−0.07 ± 0.29	0.13
	Intervention	101	0.68 ± 0.29	0.67 ± 0.28	0.70	−0.01 ± 0.27	
SF-12 PCS score	Control	88	44.0 ± 10.4	39.3 ± 13.1	<0.01	−4.7 ± 9.8	0.72
	Intervention	107	44.8 ± 9.5	40.7 ± 11.2	<0.01	−4.2 ± 10.2	
SF-12 MCS score	Control	88	53.6 ± 9.1	51.6 ± 10.5	0.14	−1.9 ± 10.8	0.56
	Intervention	107	52.4 ± 10.6	51.7 ± 9.2	0.25	−1.0 ± 11.1	
No fall	Group	N	Baseline	Follow-up	*p*-values*	Change	*p*-values**
EQ-5D utility score	Control	169	0.81 ± 0.19	0.80 ± 0.22	0.27	−0.02 ± 0.16	0.08
	Intervention	180	0.77 ± 0.24	0.80 ± 0.23	0.44	0.02 ± 0.16	
SF-12 PCS score	Control	172	47.3 ± 9.6	43.9 ± 10.4	<0.01	−3.5 ± 7.8	0.01
	Intervention	178	46.1 ± 9.6	44.5 ± 10.2	<0.01	−1.5 ± 7.1	
SF-12 MCS score	Control	172	53.1 ± 9.0	53.0 ± 8.5	0.76	−0.1 ± 9.2	0.46
	Intervention	178	53.9 ± 8.8	53.0 ± 8.9	0.40	−0.9 ± 8.8	

*C* control, *I* intervention. Data are given as mean values ± standard deviation

*Wilcoxon Signed Rank test

**Two-way ANOVA

### Cost-utility

The mean QALY difference between both groups was 0.05 QALY (gained by the intervention group) over the trial period. For the total fall-related healthcare costs (with and without the intervention costs of €120), no significantly differences between both study groups could be detected. Therefore, no incremental cost-utility ratio was calculated.

## Discussion

This is the first cost-utility analysis comparing a structured medication assessment including withdrawal of FRIDs versus ‘care as usual’ in community-dwelling older fallers conform the PROFANE guideline. The savings in fall-related healthcare cost did not differ significantly between the control and intervention group. However, the control group reported a significantly greater decline in HRQoL during the 12-months follow-up as measured with the EQ-5D utility score than the intervention group.

Various studies have reported costs and cost-effectiveness data regarding falls prevention trials with varying results. But these studies evaluated, in most cases, multifactorial interventions [30, 49–58]. One study reported on the cost-effectiveness of FRIDs withdrawal as a single intervention, and reported significant national cost savings [36]. In the current study, the savings in fall-related healthcare related costs in the intervention group did not differ significantly from usual care. This seems consistent with our findings that FRIDs-withdrawal was not effective in reducing falls [48]. There are several possible explanations for this lack of fall incidence reduction. In short, since in the last decade falls prevention guidelines have been incorporated into usual care, this may well have blunted the effect of the current intervention. In addition, a large proportion of the participants was not compliant to the intervention, especially with respect to withdrawal of psychotropic drugs (FRIDs withdrawal failed for 48 % [48]). Higher compliance rates might have led to reduced falls and lower related healthcare costs, and increased savings due to reduced medication costs (mean reduction of €38 per participant in this study). Furthermore, a less costly method of FRIDs withdrawal could be accomplished by having the GP perform the intervention. This approach has been shown to be successful, but would require an initial training programme for the GPs [59].

Until now, only one falls prevention trial reported HRQoL as recommended by ProFaNE. This multifactorial intervention trial reported no significant change in EQ-5D and SF-12 scores between the intervention and control group [18]. Four other trials used varying methods to measure HRQoL. Two found no difference in SF-36 score between the intervention and control group [24, 25]. Another multifactorial falls prevention trial, which used the 15D instrument, concluded that the intervention produced positive effects in some dimensions of HRQoL [27]. Still another trial used the World Health Organization Quality of Life instrument (WHOQoL) and measured higher quality of life in an exercise training intervention group of patients who had recently fallen [26].

Except for a structured medication assessment, including the withdrawal of FRIDs, both groups received identical care. Furthermore, withdrawal of certain commonly prescribed FRIDs such as benzodiazepines, antidepressants and opiates [38], could have resulted in lower quality of life scores in the intervention group. Nevertheless, in this study the withdrawal of FRIDs did not lower the HRQoL. Remarkably, in the secondary analysis comparing the participants without a fall during follow-up, the intervention group had less decline in the SF-12 PCS score than the control group. The fact that the intervention did not lower the HRQoL and possibly even improved it, is on its own an important outcome. The participants who fell during follow-up had significantly lower EQ-5D and SF-12 PCS scores at baseline. This is in a group of community-dwelling older persons who all visited the ED due to a fall; those who fell during follow-up had lower quality of life scores ahead of the recurrent fall. This finding has not been reported before and can be used as a tool in further research and investigations to identify those older fallers most at risk of a further fall.

An important finding in this study was the lower baseline EQ-5D utility score in the intervention group compared to the control group, regardless of similar baseline characteristics including age, gender, and number of comorbidities. This cannot be a result of differences in reporting procedures, as the method and timing of HRQoL questionnaire completion were identical for the control and intervention groups. A possible explanation for the lower baseline EQ-5D utility score in the intervention group could be the presence of more severe injuries in the intervention group at baseline. However, the injuries sustained by the participants at baseline did not differ significantly between the two groups. Overall, 42 % of participants sustained a fracture at baseline, 43 % in the control group and 40 % in the intervention group. Furthermore, 3 % of participants in the control and in the intervention groups sustained a traumatic brain injury at baseline.

Some limitations should be taken into account when interpreting results of this study.

First, recruiting participants proved challenging, the recruitment-period lasted four years despite enrolling at six hospitals. Reasons for refusing to participate have been reported previously, i.e., mobility impairment and lack of transportation options [60]. Second, the dropout of 32 participants during the 12 months follow-up might be due to the selected study population, which had a high risk of falling. These participants had often mobility impairments and other multiple morbidity which may have resulted in a refusal to continue participating in the study and visit the outpatient research clinic after 12 months follow-up. Thus the most at-risk and frail participants may have been excluded from the analysis. More individuals were excluded from the intervention group because of poorer HRQoL (23 of the 32 dropouts) which may have influenced, at least in part, the better outcomes in the intervention group.

However, the randomization would have equally divided these patients across the intervention and control group. Third, the SF-12 has been evaluated for use in large group comparisons, this may not be justified for the secondary analyses comparing participants with and without a fall during follow-up [44]*.* Fourth, randomization did not seem to balance the groups on some important variables (e.g., rehabilitation, nursing home care), which can be attributed to the relatively small sample size of the study.

## Conclusions

In the present study withdrawal of FRID’s in older persons who visited an Emergency Department due to a fall, did not lead to reduction of total health-care costs. The mean cost of the FRIDs intervention was €120 per patient, but this did not result in significant cost reductions in total healthcare costs. However, the withdrawal of FRIDs reduced medication costs with a mean of €38 per participant which in combination with less decline in HRQoL is an important result.

## Additional file

Additional file 1: Improvefall study patient questionnaire. Patients included in the Improvefall studie received a patient outcome questionnaire regarding medical history, quality of life and a fall risk profile. (PDF 536 kb)

## Abbreviations

ED: Emergency department
EQ-5D: EuroQol-5D
FRIDs: Fall-risk-increasing-drugs
HRQoL: Health-related quality of life
IMPROveFALL: Improving medication prescribing to reduce risk of FALLs
MCS: Mental component summary score
MMSE: Mini-mental state examination
PCS: Physical component summary score
ProFaNE: Prevention of falls network Europe
QALY: Quality-adjusted life year
SF-12: Short form-12
WHOQoL: World health organization quality of life instrument
