# Supplementary material for: Cost-utility of medication withdrawal in older fallers: results from the improving medication prescribing to reduce risk of FALLs (IMPROveFALL) trial
Source: BMC Geriatr. 2016 Nov 4;16:179. doi: 10.1186/s12877-016-0354-7 (PMC5096283; doi:10.1186/s12877-016-0354-7)
Supplement: Additional file 1: — Improvefall study patient questionnaire. Patients included in the Improvefall studie received a patient outcome questionnaire regarding medical history, quality of life and a fall risk profile. (PDF 536 kb) [file 12877_2016_354_MOESM1_ESM.pdf]

IMPROVEFall

Erasmus MC Universitair Medisch Centrum Rotterdam

VU medisch centrum

First visit

VU medisch centrum

IMPROVEFall

Application form geriatrics outpatient clinic:

Reference through the clinical trial, after fall incident in Emergency Room

Date of registration	:
Is patient known in VUmc?	:
Patient number	:
Date of birth	:
Name	:
Address	:
Zipcode/city	:
Phone #	:
General practitioner	:
Phone # general practitioner	:
Pharmacy	:
Phone # pharmacy	:

- A comprehensive medical history + medication list has to be retrieved from the general practitioner.
- A current medication list has to be retrieved from the pharmacy.

Will be mailed	: to do / done
Will be faxed (020-4440505)	: to do / done

Date of outpatient clinic visit	:
Time of outpatient clinic visit	:

Written confirmation concerning the appointment in the outpatient clinic:

Date letter was sent:

VU medisch centrum

IMPROVEFall

Addendum: Geriatrics outpatient clinic status

1. EuroQol-5D

(Original state, copy on behalf of CRF)

2. Short Form 12

(Original state, copy on behalf of CRF)

3. Fall risk profile

(Original state, copy on behalf of CRF)

4. Country of birth patient ……………………..
   Country of birth father ……………………..
   Country of birth mother ……………………..

5. Pharmacy information obtained and attached after 2nd visit: to do / done
   General practitioner information obtained and attached after 2nd visit: to do / done

6. Physical examination

Height	…….. cm
Weight	……. kg
Calf circumference	Left …,. / …,. Right …,. / …,..cm
Upper arm circumference	Left …,. / …,. Right …,. / …,..cm
Handgrip strength	Left …,. / …,. Right …,. / …,..cm

7. Tests

- Timed get up and go test……………………/.........................sec
- Getting up from a chair 5 times in a row (time it……………………/.........................sec (crossed arms in front of chest)
- Hold tandem stance for 10 seconds..............................perfect/divergent

8. Laboratory (blood work) research for all patients

Sodium	mmol/L	ASAT	U/l
Potassium	mmol/L	ALAT	U/l
Creatinine	umol/L	Hb	mmol/L
Urea	U/l	Ht	
Glucose	mmol/l	25-hydroxy vitamin D	nmol/L

Please note: For the purpose of DNA analysis, a collection tube with 4-cc EDTA blood will be taken. This will be send to Ee-575 with the attached inquiry form.

9. Additional research

- ECG: if indication, dependent on medication change
- X-thorax: if indication, dependent on medication change
- 24-hour Holtertape: if indication, dependent on medication change

Copy geriatrics outpatient clinic status on behalf of CRF!

VU medisch centrum

IMPROVEFall

EQ-5D

Follow-up: ..... 2 weeks ..... 1 year

Patiënt ID number | . . | | | |

Date completed ... ... / ... ... / 200 ...

Your own health state today

By placing a tick in one box in each group below, please indicate which statement best describes your own health state today.

Do not tick more than one box in each group.

Mobility

I have no problems in walking about ☐
I have some problems in walking about ☐
I am confined to bed ☐

Self-care

I have no problems with self-care ☐
I have some problems washing and dressing myself ☐
I am unable to wash and dress myself ☐

Usual activities (eg. work, study, housework, family or leisure activities)

I have no problems with performing my usual activities ☐
I have some problems with performing my usual activities ☐
I am unable to perform my usual activities ☐

Pain/discomfort

I have no pain or discomfort ☐
I have moderate pain or discomfort ☐
I have extreme pain or discomfort ☐

Anxiety/depression

I am not anxious or depressed ☐
I am moderately anxious or depressed ☐
I am extremely anxious or depressed ☐

Your own health state today

To help people say how good or bad a health state is, we have drawn a scale (rather like a thermometer) on which the best state you can imagine is marked 100 and the worst state you can imagine is marked 0.

We would like you to indicate on this scale how good or bad your own health is today, in your opinion. Please do this by drawing a line from the box below to whichever point on the scale indicates how good or bad your health state is.

Your own health state today

Best imaginable health state

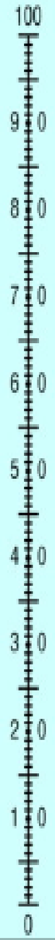

Worst imaginable health state

SF-12 Health Survey

This survey asks for your views about your health. This information will help keep track of how you feel and how well you are able to do your usual activities. **Answer each question by choosing just one answer**. If you are unsure how to answer a question, please give the best answer you can.

1. **In general, would you say your health is**:

☐1 Excellent ☐2 Very good ☐3 Good ☐4 Fair ☐5 Poor

The following questions are about activities you might do during a typical day. Does your health now limit you in these activities? If so, how much?

	**YES, limited a lot**	**YES, limited a little**	**NO, not limited at all**
2. **Moderate activities** such as moving a table, pushing a vacuum cleaner, bowling, or playing golf.	☐1	☐2	☐3
3. Climbing **several** flights of stairs.	☐1	☐2	☐3

During the past 4 weeks, have you had any of the following problems with your work or other regular daily activities as a result of your physical health?

	**YES**	**NO**
4. **Accomplished less** than you would like.	☐1	☐2
5. Were limited in the **kind** of work or other activities.	☐1	☐2

During the past 4 weeks, have you had any of the following problems with your work or other regular daily activities as a result of any emotional problems (such as feeling depressed or anxious)?

	**YES**	**NO**
6. **Accomplished less** than you would like.	☐1	☐2
7. Did work or activities **less carefully than usual**.	☐1	☐2

8. **During the past 4 weeks, how much did pain interfere with your normal work (including work outside the home and housework)?**

☐1 Not at all ☐2 A little bit ☐3 Moderately ☐4 Quite a bit ☐5 Extremely

These questions are about how you have been feeling during the past 4 weeks.
For each question, please give the one answer that comes closest to the way you have been feeling.

How much of the time during the past 4 weeks…

	All of the time	Most of the time	A good bit of the time	Some of the time	A little of the time	None of the time
9. Have you felt calm & peaceful?	☐1	☐2	☐3	☐4	☐5	☐6
10. Did you have a lot of energy?	☐1	☐2	☐3	☐4	☐5	☐6
11. Have you felt down-hearted and blue?	☐1	☐2	☐3	☐4	☐5	☐6

12. **During the past 4 weeks, how much of the time has your physical health or emotional problems interfered with your social activities (like visiting friends, relatives, etc.)?**

☐1 All of the time ☐2 Most of the time ☐3 Some of the time ☐4 A little of the time ☐5 None of the time

Patient name: Date: PCS: MCS:

Visit type (circle one)
Preop 6 week 3 month 6 month 12 month 24 month Other:_________

FOR WEB-ONLY PUBLICATION

Appendix The LASA Fall Risk Profile as Developed in the Longitudinal Aging Study Amsterdam (LASA) [10] and Validated in the Fall Prevention Trial.

LASA Fall Risk Profile **Score**

1. How often did you fall during the past 12 months, including the last fall?

☐ <2 falls in the past 12 months (0 points)

☐ ≥2 falls in the past 12 months (4 points) …… points

2. Do you often have dizzy spells?

☐ no (0 points)

☐ yes (4 points) …… points

3a. Are you able to use your own method of transport or public transportation?

☐ no (go to question 3b)

☐ yes (0 points, go to question 4)

3b. Are you able to go up 15 steps without standing still?

☐ no (go to question 3c)

☐ yes (0 points, go to question 4)

3c. Are you able to cut your own toenails?

☐ no (3 points when all three questions are answered with “no”)

☐ yes (0 points, go to question 4) …… points

4. Grip strength right hand

....………….. kg (1)

....………….. kg (2)

Maximum grip strength right hand: ....………….. kg

Grip strength left hand

....………….. kg (1)

....………….. kg (2)

Maximum grip strength left hand: ....………….. kg

—————— +

Total maximum hand grip strength (TMG) ....………….. kg

female (TMHG):	male (TMHG):	
>32 kg (0 points)	>56 kg (0 points)	
≤32 kg (3 points)	≤56 kg (3 points)	…… points

5. Body weight (without shoes)

....………….. kg

female:	male:	
>62 kg (0 points)	>70 kg (0 points)	
≤62 kg (2 points)	≤70 kg (2 points)	…… points

6. Do you have a dog or a cat?

☐ no (0 points)

☐ yes (2 points) …… points

7. How concerned are you that you might fall when:

	not at all	somewhat	fairly	very
	0	1	2	3
cleaning the house?	☐	☐	☐	☐
getting dressed and undressed?	☐	☐	☐	☐
preparing simple meals?	☐	☐	☐	☐
taking a bath or shower?	☐	☐	☐	☐
going to the shop?	☐	☐	☐	☐
getting in or out of a chair?	☐	☐	☐	☐
going up or down stairs?	☐	☐	☐	☐
walking around outside?	☐	☐	☐	☐
reaching up or bending down?	☐	☐	☐	☐
answering the telephone?	☐	☐	☐	☐

Per question 0-3 points can be scored

Add up all the points: <1 (0 points)

≥1 (2 points) …… points

8. Do you sometimes drink alcohol?

☐ no (0 points, go to question 9)

☐ yes

Do you drink alcohol every day? yes/no

How many days per week do you drink alcohol? …… days/week

How many glasses of alcohol do you drink each time?

- during the week: …… glasses
- during the weekends: …… glasses

Total number of glasses per week: …… glasses/week

(more than 15 glasses of alcohol per week = 1 point) …… points

9. What is the highest level of education that you completed with a certificate?

☐ primary school (0 points)

☐ equivalent to junior high school, apprenticeship (0 points)

☐ high school, college, university (1 point)

One point is scored if 11 or more years of education have been completed. …… points

10. Item 1: ≥2 falls in the past 12 months **AND** Item 7: ≥3 points (add 4 points) …… points

11. Item 8: >15 glasses of alcohol **AND** Item 9: ≥11 years of education (add 4 points) …… points

\+

Total score: …….. points

VU medisch centrum

IMPROVEFall

Patient history questions

Fall incident date …./…./20…. during (**activity**) (**location)** ______________________________

____

____

____

____

____

____

____

Amount of fall incidents in the past 12 months

Dizziness	Yes/No*
Lightheaded	Yes/No*
Headaches	Yes/No*
Orthostasis complaints	Yes/No*
Twitching	Yes/No*
Tongue bite	Yes/No*
Loss of consciousness	Yes/No*
Fainting	Yes/No*
Balance problems	Yes/No*
Chest pains	Yes/No*
Palpitations	Yes/No*
Polyneuropathy	Yes/No*
Bad sight	Yes/No*

*cross out what is not applicable

VU medisch centrum

IMPROVEFall

Scale for activities of daily living (ADL) according to KATZ

0 = independent, 1 = some assistance, 2 = full assistance

Item	Score		
Bathing and showering	0	1	2
Dressing	0	1	2
Toilet hygiene	0	1	2
Functional mobility (get in and out of bed, and get into and out of a chair)	0	1	2
Self-feeding	0	1	2
Continence (0, <2wk incontinence = 1, incontinence = 2)	0	1	2

Total ___/ 12

Scale of I (instrumental) ADL (by Lawton & Brody)

0 = independent, 1 = some assistance, 2 = full assistance

Item	Score		
Ability to use telephone	0	1	2
Mode of transportation (to destinations beyond walking distance)	0	1	2
Shopping	0	1	2
Food preparation (hot meals)	0	1	2
Housekeeping	0	1	2
Responsibility of own medications	0	1	2
Ability to handle finances	0	1	2

Total ___/ 14
